# Supplementary material for: Patterned human microvascular grafts enable rapid vascularization and increase perfusion in infarcted rat hearts
Source: Nat Commun. 2019 Feb 4;10:584. doi: 10.1038/s41467-019-08388-7 (PMC6362250; doi:10.1038/s41467-019-08388-7)
Supplement: Supplementary file 1 — Supplementary Information [file 41467_2019_8388_MOESM1_ESM.pdf]

Supplementary Materials

Patterned human microvascular grafts enable rapid vascularization and increase perfusion in infarcted hearts

Meredith A. Redd, Ph.D [1-3#], Nicole Zeinstra[1-3#], Wan Qin, Ph.D [1], Wei Wei [1], Amy Martinson [2-4], Yuliang Wang[3,5], Ruikang K. Wang, Ph.D [1], Charles E. Murry, M.D., Ph.D.[1-4,6*], Ying Zheng, Ph.D.[1-3*]

[1]Department of Bioengineering, [2]Center for Cardiovascular Biology, [3]Institute for Stem Cell and Regenerative Medicine, [4]Department of Pathology, [5] Paul G. Allen School of Computer Science & Engineering, [6]Department of Medicine/Cardiology, University of Washington, Seattle, WA 98109.

[#] These authors contributed equally to this work.

* Corresponding authors:

Ying Zheng, PhD., yingzy@uw.edu

Charles E. Murry, PhD., murry@uw.edu

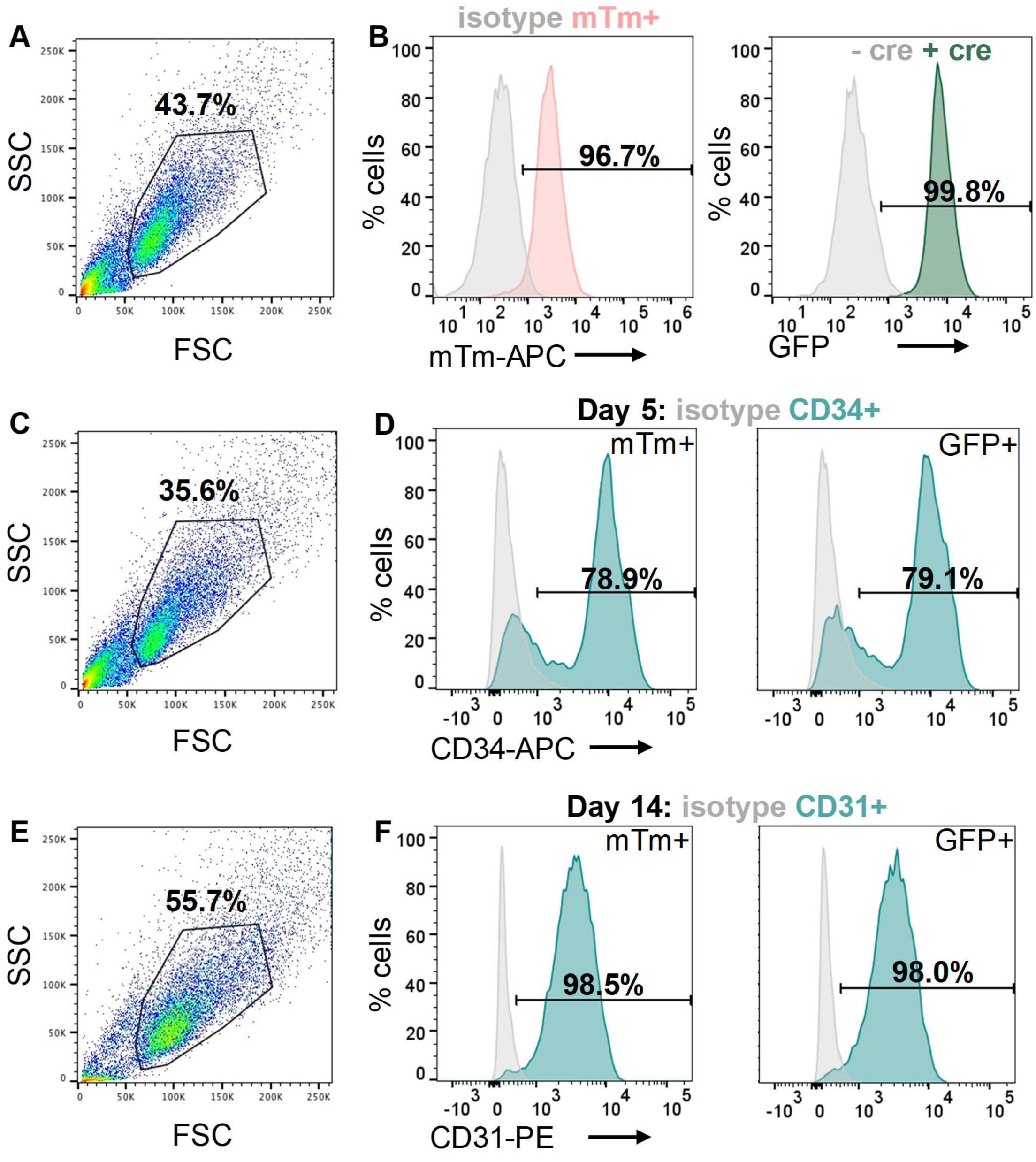

Supplementary Figure 1. Flow cytometry of hESC-ECs from mTm- and GFP-expressing hESCs (A-B) Assessment of TdTomato expression in dual fluorescent hESC reporter line with GFP expression after Cre recombinase-induced recombination and purification. (A) Forward and side scatter used to gate main population. (B) mTm expression (DsRed+, left) before and GFP expression (right) after Cre recombinase-induced recombination and purification with overlay of isotype controls. (C-D) Assessment of Day 5 CD34+ progenitor cells. (C) Forward

and side scatter used to gate main population. (D) CD34 expression of mTm-hESC (left) and GFP-hESC (right) differentiations with overlay of isotype controls. (E-F) Assessment of Day 14 CD31+ populations. (E) Forward and side scatter used to gate main population. (F) CD31 expression of mTm-hESC-ECs (left) and GFP-hESC-ECs (right) with overlay of isotype controls.

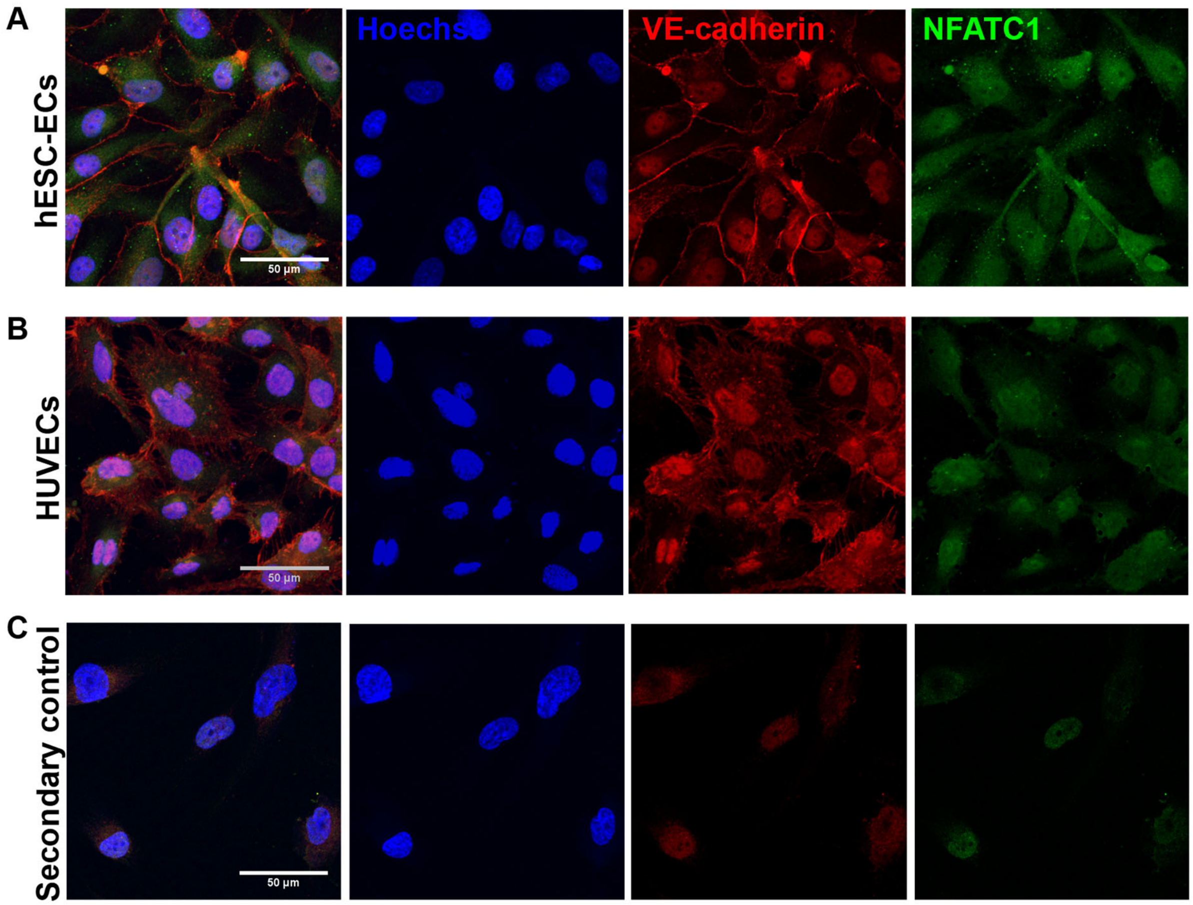

Supplementary Figure 2. Additional characterization of hESC-ECs by NFATC1 staining (A-C) Immunofluorescence images of endothelial cells stained for VE-cadherin (red) and NFATC1 (green). Scale bar, 50 µm. (A) hESC-ECs in culture. (B) HUVECs in culture. (C) Staining control with secondary antibody only. Representative images for (A), (B), and (C) from 2 biological replicates for each condition, with similar results. Hoechst-stained nuclei, blue.

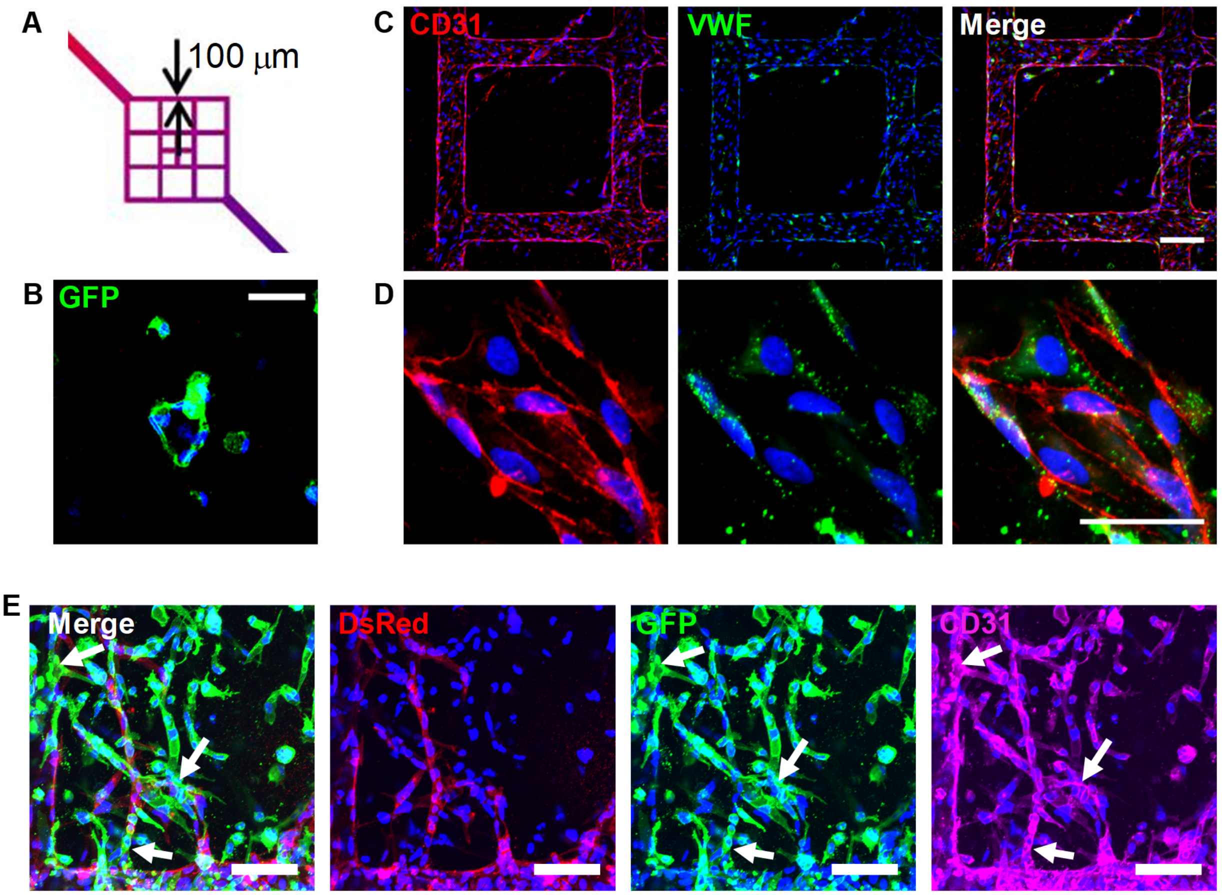

Supplementary Figure 3. Additional characterization of hESC-ECs in engineered microvessels (A) Square network geometry that is used to mold microchannels for all *in vitro* experiments. The vessel diameter is 100 μm in the network, the distance between parallel vessels are 300 μm in the center and 700 μm in other surrounding regions. (B) Confocal z-slice of a *de novo* GFP-hESC-EC lumen formed in the bulk collagen. Scale bar, 40 μm. (C-D) Maximum intensity projection (MIP) of confocal z-stack of μV only construct cultured for 4 days and stained for CD31 (red, left) and VWF (green, middle). Merged images, right. (C) 10X view of patterned microvessel to show global expression throughout the construct. Scale bar, 200 μm. (D) High magnification image of an endothelial sprout from the patterned vessel. Scale bar, 50 μm. (E) MIP of confocal z-stack of tube connections (white arrows) between *de novo* GFP-hESC-EC lumen in the bulk collagen and the mTm-hESC-EC seeded microvessels and sprouts stained for

DsRed (red, middle left), GFP (green middle right), and CD31 (magenta, right). Merged image, left. Scale bar, 100 μm. Representative images for (B) and (E) from 7 biologically independent samples of D7 μV +SA, with similar results. Representative images for (C) and (D) from 2 biologically independent samples for D4 μV only, with similar results. Hoechst-stained nuclei, blue. EC, endothelial cell. D4, after 4 days of culture. D7, after 7 days of culture.

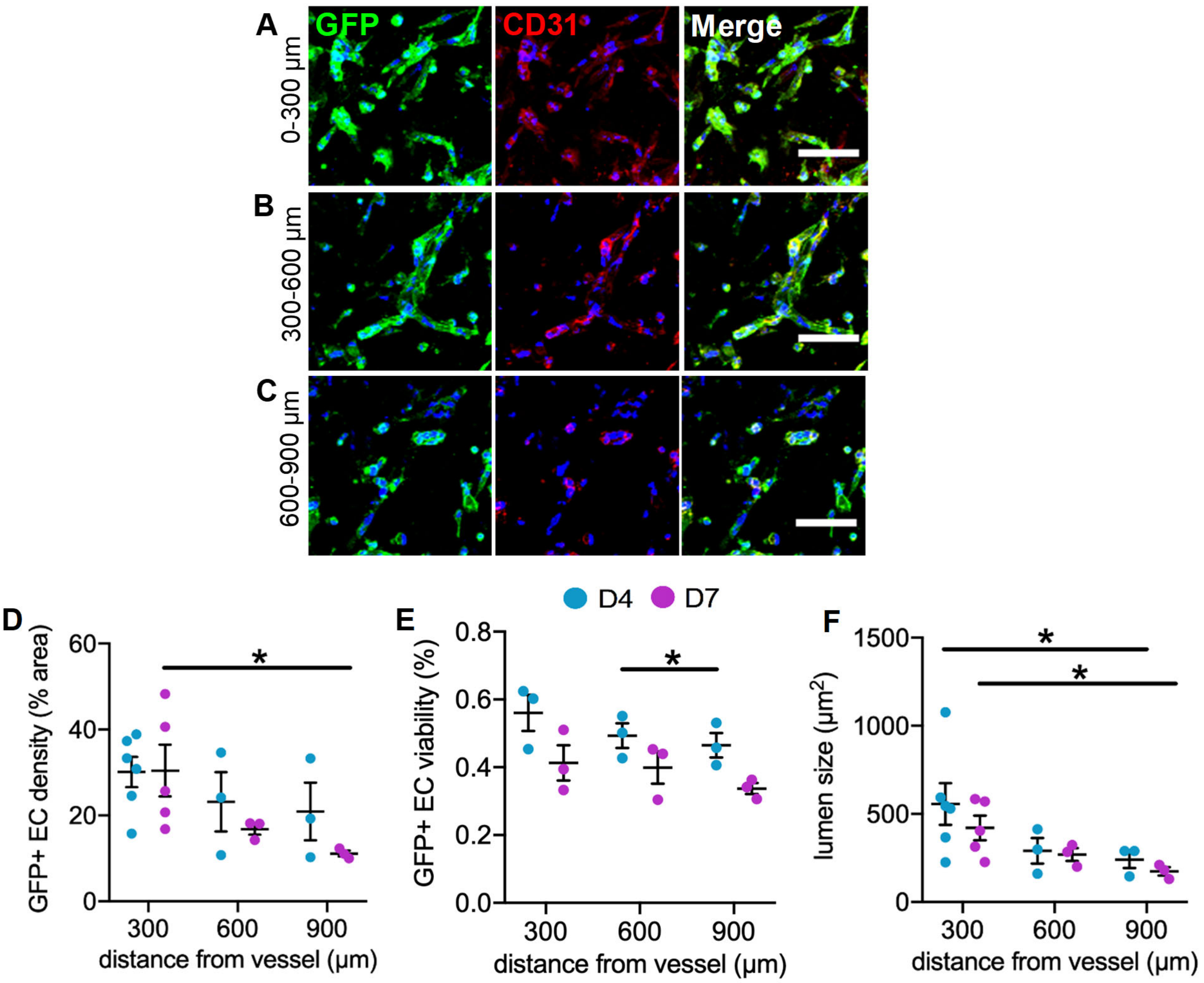

Supplementary Figure 4. Additional characterization of hESC-ECs in bulk collagen in engineered microvessels (A-C) Maximum intensity projection of confocal z-stack of GFP-hESC-EC tubulogenesis in interstitial collagen matrix of µV + SA device after 7 days of culture at distances 0-300 µm (A), 300-600 µm (B), and 600-900 µm (C) from the patterned microvessel wall stained for GFP (green, left) and CD31 (red, middle). Merged image, right. Scale bar, 100 µm. (D-F) Quantification of GFP-hESC-EC *de novo* lumens in relation to proximity to the patterned vessel in µV + SA constructs after 4 days (blue circles) and 7 days (purple circles) of culture. Data were quantified in for each construct at three distances from the patterned microvessel wall: 0-300 µm, 300-600 µm, and 600-900 µm (x-axis). (D) Quantification of GFP+ EC density (percent area occupied by GFP+ ECs). N = 6,5 biologically independent samples for

D4 and D7, respectively. P = 0.032 for D4 at 0-300 μm and 600-900 μm, $p > 0.05$ for all others (two-tailed t-test). (E) Quantification of cell viability (as percentage of GFP+ ECs) in the bulk matrix. N = 3 biologically independent samples for both D4 and D7. P = 0.046 for 300-600 μm and 600-900 μm for D4, $p > 0.05$ for all others (paired t-test). (F) Quantification of lumen size for *de novo* lumens. N = 6,5 biologically independent samples for D4 and D7, respectively. With data only available for 3,2 constructs at 0-300 μm. P = 0.022 for D4 at 0-300 μm and 600-900 μm, p = 0.046 for D4 at 0-300 μm and 600-900 μm, $p > 0.05$ for all others (two-tailed t-test). Representative images for (A), (B), and (C) from 7 biologically independent samples of D4 μV +SA, with similar results. Hoechst-stained nuclei, blue. Error bars, mean ± SEM. *$p < 0.05$ determined using two-tailed t-test for (E) and (G) and paired t-test for (F). EC, endothelial cell. D4, after 4 days of culture. D7, after 7 days of culture.

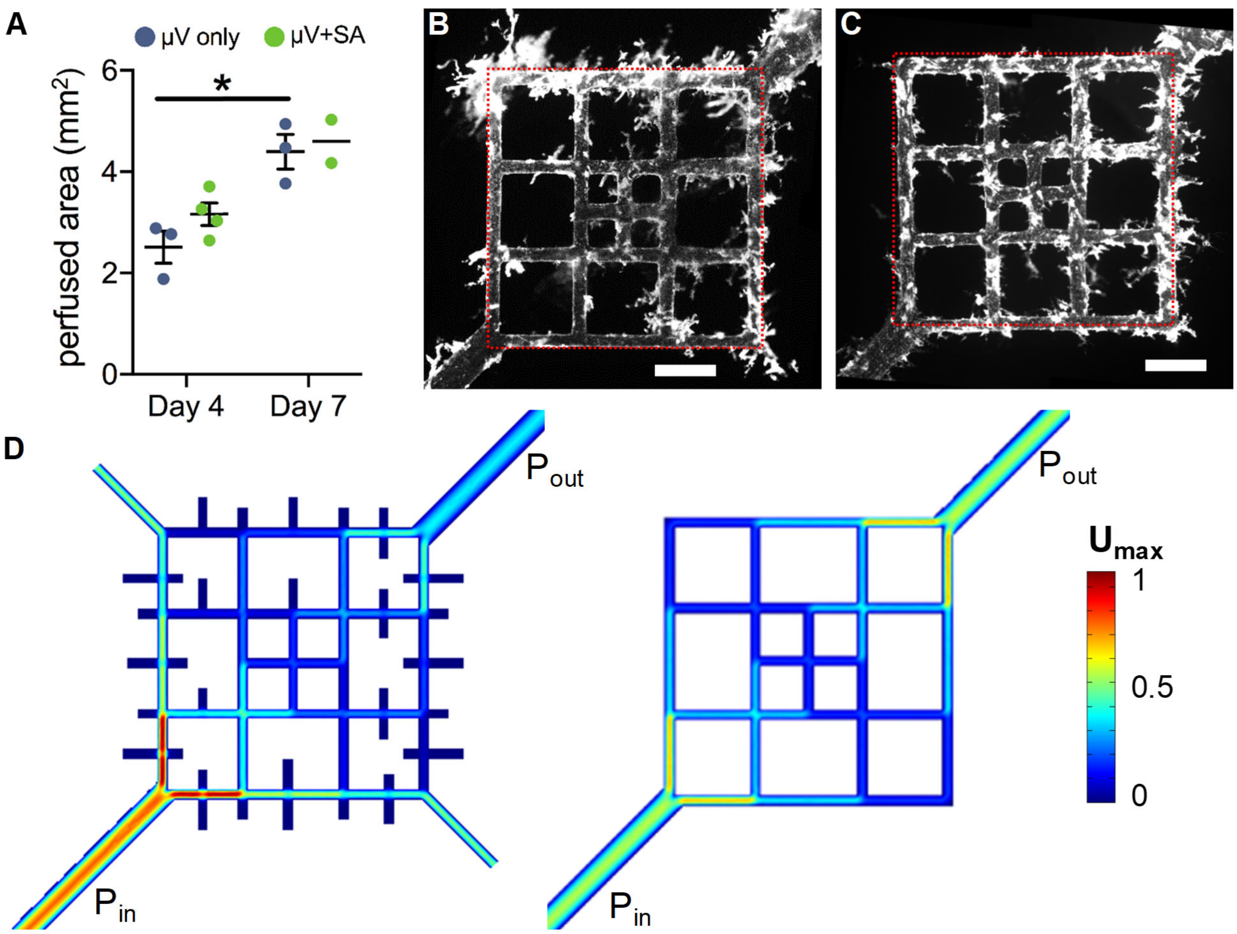

Supplementary Figure 5. Perfusion in engineered microvessels. (A) Quantification of perfused area in bead perfusion experiments for μV only (blue circles) and μV + SA (green circles) constructs after 4 days and 7 days of culture. N = 3,4,3,2 biologically independent samples for D4 μV only, D4 μV +SA, D7 μV only, and D7 μV +SA, respectively. p = 0.016 for D4 μV only and D7 μV only, p > 0.05 for all others (two-tailed t-test). (B-C) Large images of bead perfused μV only (B) and μV + SA (C) constructs. For each vessel, the stitched image encompassed a field of view twice the area of the original pattern boundary (red dashed line) or 40% greater in each direction. Scale bar, 500 μm. (D) Flow simulation using COMSOL software for pressure-driven steady-state laminar flow through a square network model either with sprouts (left) or without sprouts (right). Newtonian fluid properties of water and pressure drop of 100 Pa between inlet and outlet were assumed. Model with sprouts has 25% greater area than model

without sprouts. For colormap, flow velocity normalized by the maximal velocity (U_{max}) in both networks. The maximal velocity was approximately 25% higher in sprouted vessel model than the original vessel model. P_{in}, inlet pressure. P_{out}, outlet pressure. Representative images for (B) and (C) from 3 biologically independent samples of μV only and 2 biologically independent samples of μV + SA, with similar results. Error bars, mean ± SEM. *$p < 0.05$ determined using two-tailed t-test.

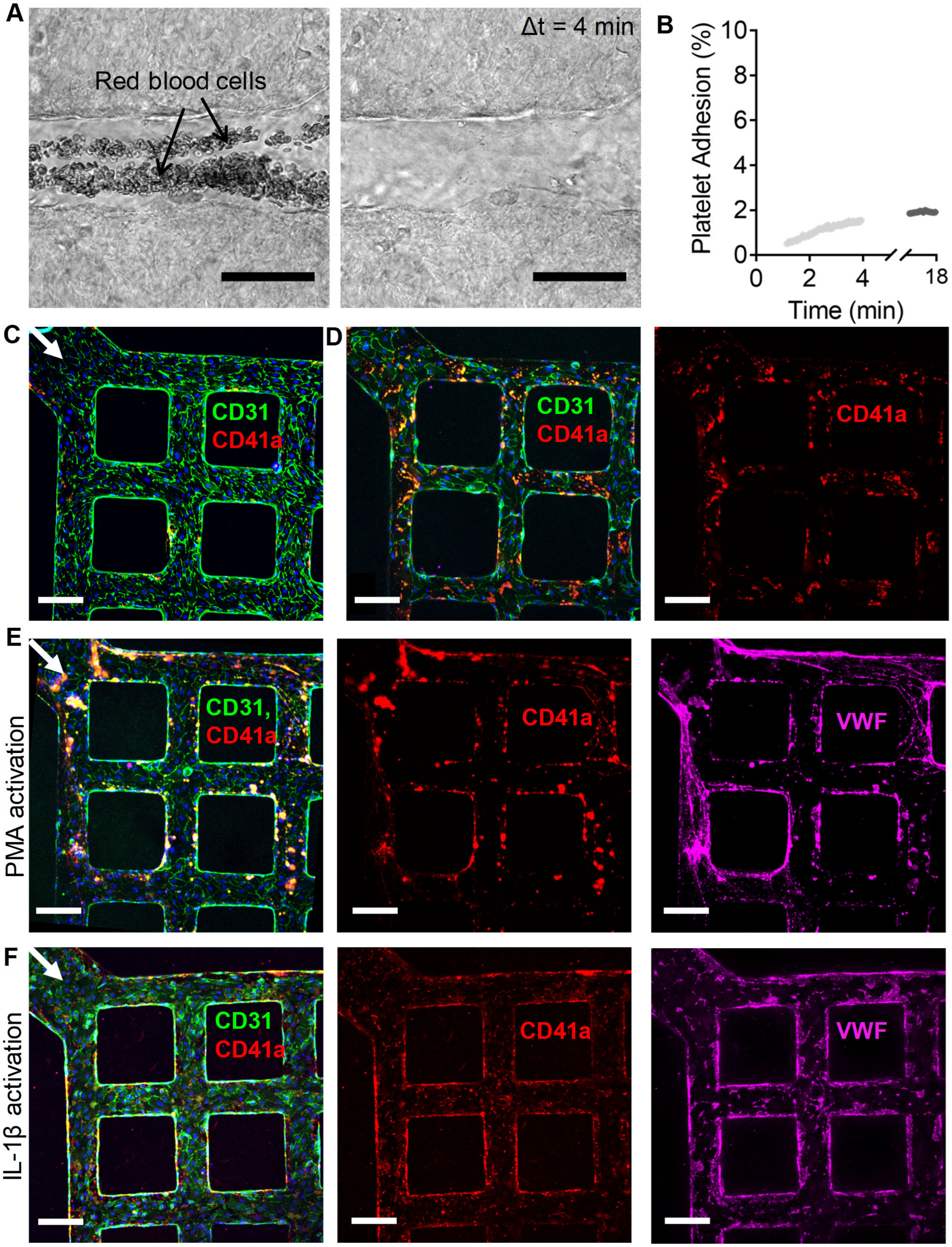

Supplementary Figure 6. Whole blood perfusion and washout of engineered microvessels (A)

Red blood cell perfusion through microvascular network (left) and red blood cell clearance after 4 minutes of PBS perfusion (right). Scale bar, 100 μm. (B) Average platelet accumulation during initial perfusion of whole blood as a percentage of the microvessel wall surface area for 18-minute perfusion. N = 3 biologically independent samples. (C-F) Maximal intensity projection of confocal z-stacks of constructs after 10 minutes of whole blood perfusion, fixed and stained for CD41a (red), CD31 (green), and VWF (magenta). Constructs seeded with HUVECs in control conditions (C), hESC-ECs in control conditions (D), hESC-ECs with 30-minute treatment of 50 ng/mL PMA prior to whole blood perfusion (E) and hESC-ECs with 30-minute treatment of 1μg/mL IL-1β prior to whole blood perfusion (F). Scale bar, 200 μm. Representative images for (A) from 3 biologically independent samples of constructs seeded with hESC-ECs in control conditions, with similar results. Representative images for (C), (D), (E), and (F) from 2,2,2,3 biologically independent samples for constructs seeded with HUVECs in control conditions, hESC-ECs in control conditions, hESC-ECs with 30-minute treatment of 50 ng/mL PMA prior to whole blood perfusion, and hESC-ECs with 30-minute treatment of 1μg/mL IL-1β prior to whole blood perfusion, respectively, with similar results. Hoechst-stained nuclei, blue.

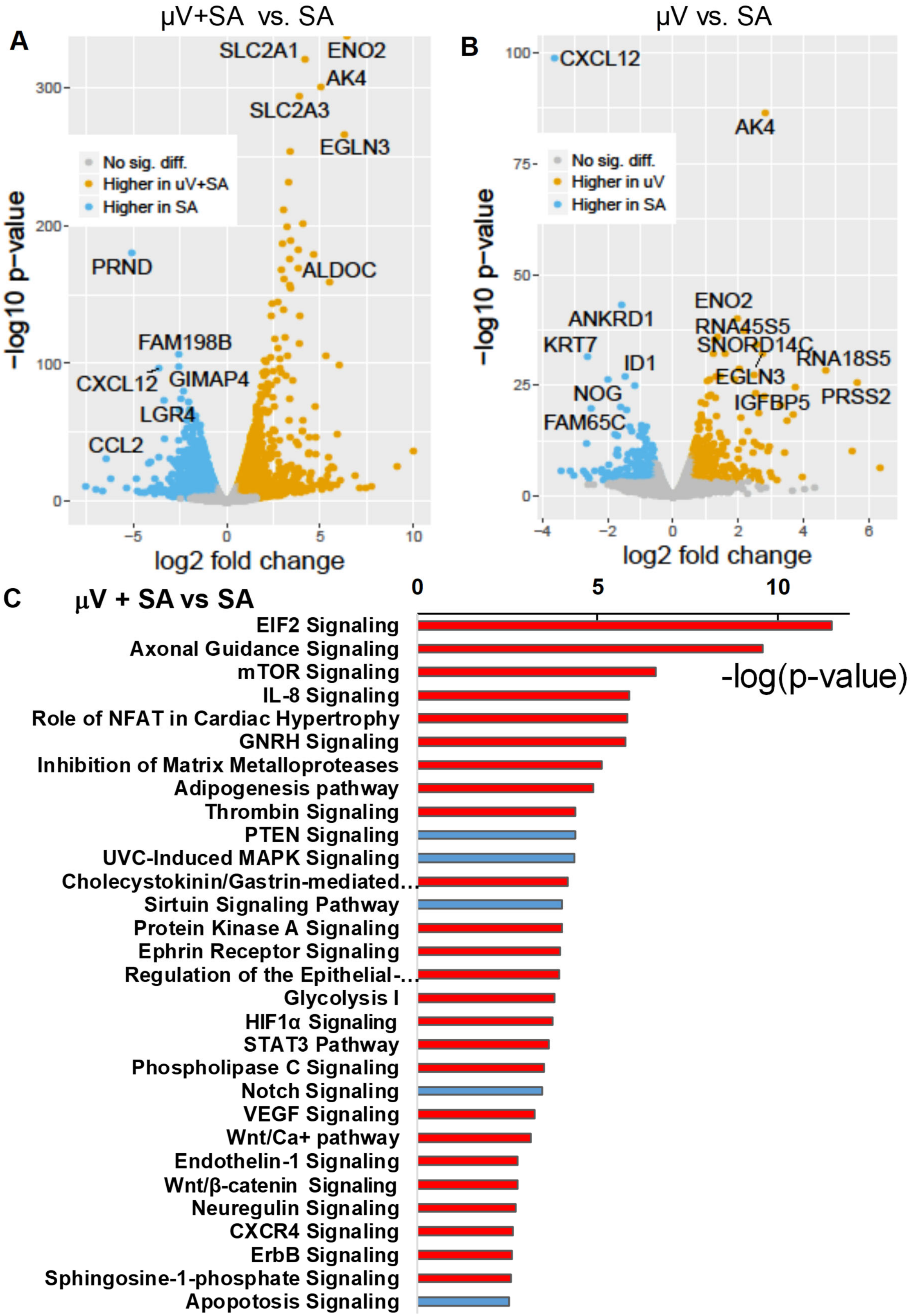

μV+SA vs. SA
A
SLC2A1
ENO2
AK4
SLC2A3
EGLN3
No sig. diff.
Higher in uV+SA
Higher in SA
PRND
ALDOC
FAM198B
CXCL12
GIMAP4
LGR4
CCL2
−log10 p−value
log2 fold change
μV vs. SA
B
CXCL12
AK4
No sig. diff.
Higher in uV
Higher in SA
ANKRD1
ENO2
RNA45S5
SNORD14C
KRT7
ID1
RNA18S5
EGLN3
NOG
IGFBP5
PRSS2
FAM65C
−log10 p−value
log2 fold change
C
μV + SA vs SA
-log(p-value)
EIF2 Signaling
Axonal Guidance Signaling
mTOR Signaling
IL-8 Signaling
Role of NFAT in Cardiac Hypertrophy
GNRH Signaling
Inhibition of Matrix Metalloproteases
Adipogenesis pathway
Thrombin Signaling
PTEN Signaling
UVC-Induced MAPK Signaling
Cholecystokinin/Gastrin-mediated…
Sirtuin Signaling Pathway
Protein Kinase A Signaling
Ephrin Receptor Signaling
Regulation of the Epithelial-…
Glycolysis I
HIF1α Signaling
STAT3 Pathway
Phospholipase C Signaling
Notch Signaling
VEGF Signaling
Wnt/Ca+ pathway
Endothelin-1 Signaling
Wnt/β-catenin Signaling
Neuregulin Signaling
CXCR4 Signaling
ErbB Signaling
Sphingosine-1-phosphate Signaling
Apoptosis Signaling

Supplementary Figure 7. Additional gene expression analysis for SA, μV only, and μV + SA constructs after 3 days of culture *in vitro* (A-B) Volcano plots showing different gene expression comparing μV + SA constructs vs. SA constructs (A) and μV only vs. SA only constructs (B). (C) Canonical pathway analysis for comparison between μV + SA and SA only constructs.

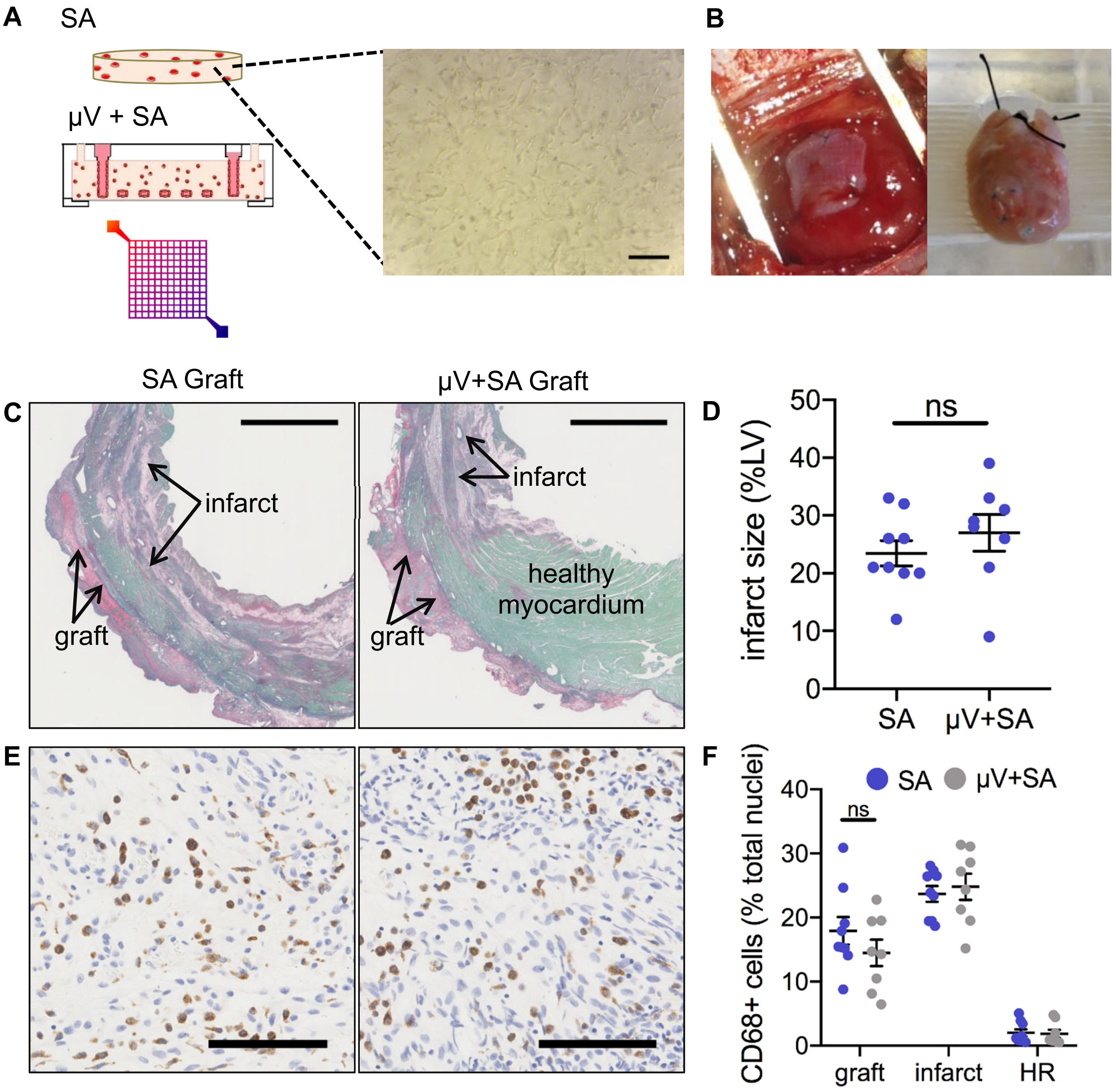

Supplementary Figure 8. Assessment of ischemia/reperfusion induced myocardial injury and macrophage infiltration of engrafted hearts (A) Schematics of SA (top) constructs and μV + SA (middle) constructs with large grid-like geometry used for network patterning (bottom). Brightfield image of SA construct after 4 days of culture just prior to implantation. Scale bar, 200 μm. (B) Images of vascular grafts sutured onto epicardial surface of the rat heart immediately after implantation surgery (left) and after heart excision, cannulation, and perfusion fixation

(right). (C) Picrosirius red/fast green stain to SA (left) and µV + SA (right) grafts. Collagenous regions, such as the infarct and grafts, stain red/purple and healthy tissue regions stain green. Scale bar, 2 mm. (D) Quantification of infarct size as a percentage of left ventricle area. N = 9 biologically independent animals for both SA and µV + SA grafts. $p > 0.05$ (two-tailed t-test). (E) CD68 (brown) immunohistochemistry with hematoxylin (blue) counterstain to detect nuclei in SA (left) and µV + SA (right) grafts. Scale bar, 100 µm. (F) Quantification of CD68+ cells as a percentage of total nuclei in the grafts, for SA (purple circles) and µV + SA (grey circles) in graft, infarct region, and healthy region (HR). N = 9,8 biologically independent animals for SA and µV + SA, respectively. $p > 0.05$ for SA and µV + SA for each group (two-tailed t-test). Representative images for (A), (C), and (E) from 9 biologically independent animals with SA grafts and 8 biologically independent animals with µV + SA grafts and 17 corresponding infarct regions and healthy regions from all animals containing grafts, with similar results. Error bars, mean ± SEM. ns = non-significant ($p > 0.05$).

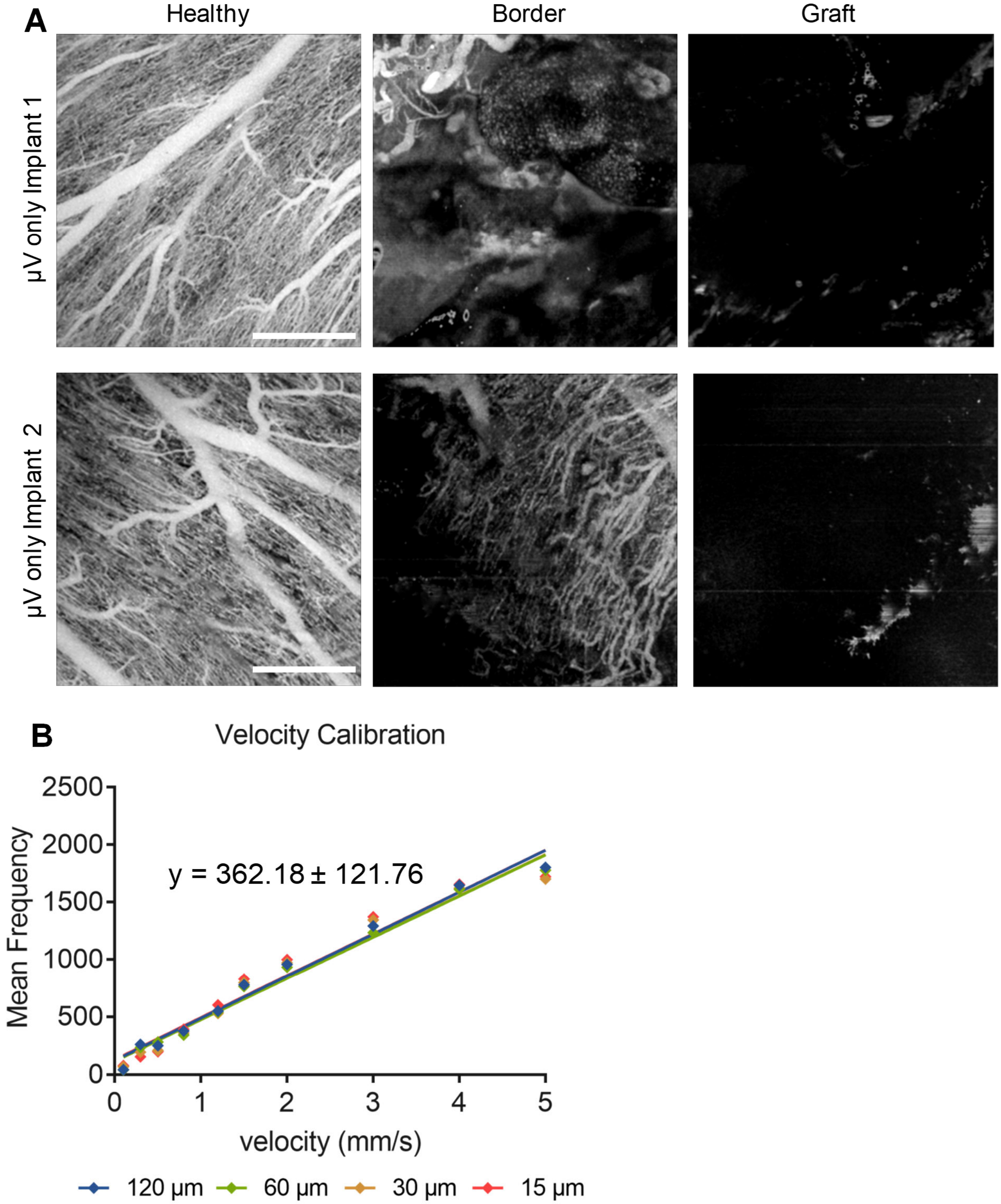

Supplementary Figure 9. Additional information for optical microangiography imaging (A) Implantation of engineered microvessels in infarcted rat heart model with optical

microangiography (OMAG) assessment of perfused structures 5 days post implantation for μV only constructs. Healthy region, left. Border between healthy and infarct region, middle. Graft region, right. Top and bottom are two independent implantation samples. Scale bar, 500 μm. (B) Calibration of OMAG mean frequency to velocity. Data was generated by acquiring velocimetry data for microfluidic channels at different size perfused at a range of velocities. The slope of the linearly correlated data was used to convert mean frequency to velocity for the Langendorff-perfused rat hearts. Representative images for (A) from 5 biologically independent animals.

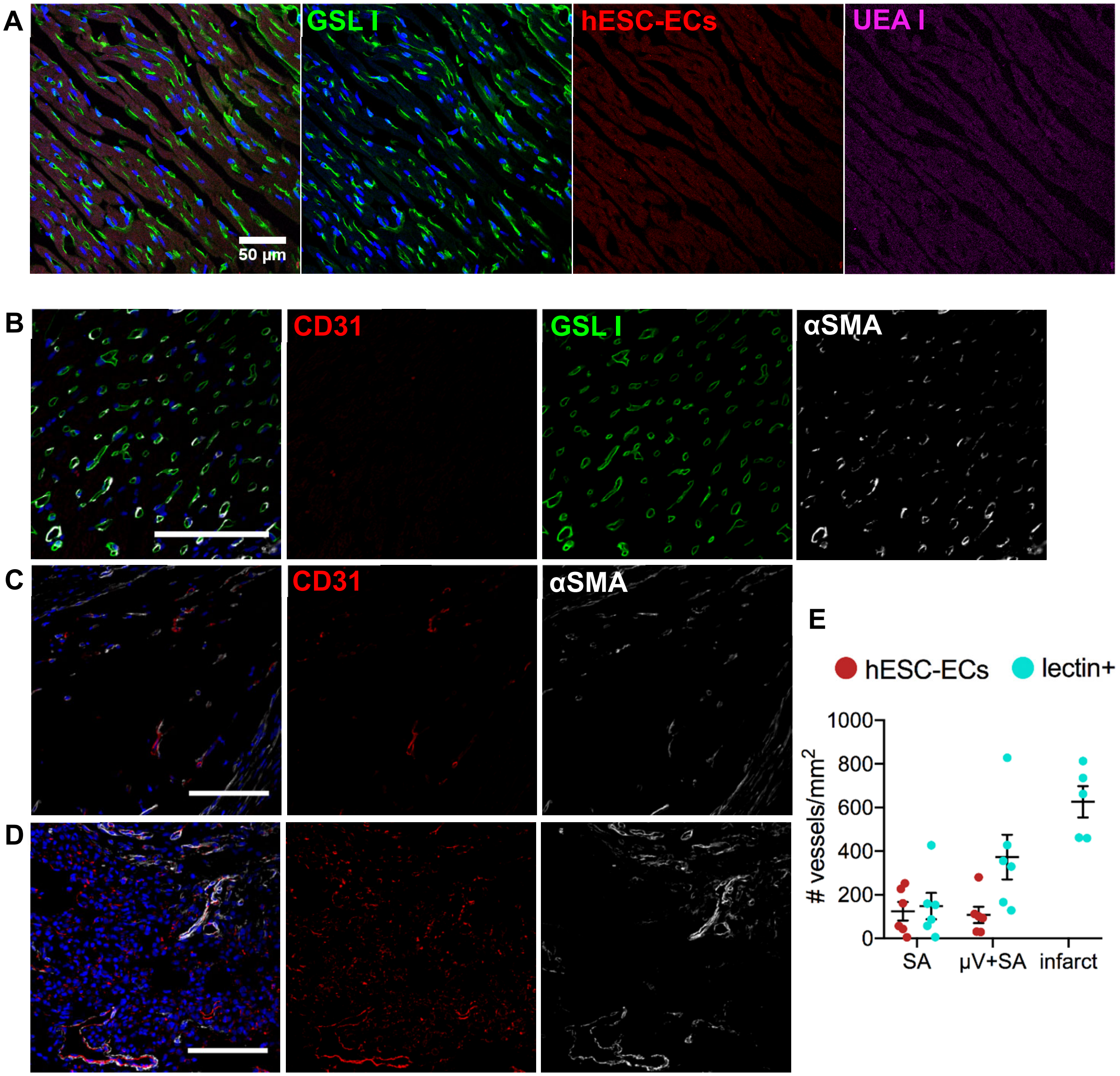

Supplementary Figure 10. Additional histological analysis for human endothelial cells and perfused vessels in grafts 5 days post implantation (A) Lectin staining controls in a rat heart for GSL I (green), mTm-hESC-ECs (red), and UEA I (magenta). Scale bar, 50 µm. (B-D) α-smooth muscle actin (αSMA)-coated human vessels identified by human CD31 expression in rat myocardium (B), SA grafts (C) and µV + SA grafts (D) stained for GSL I lectin (green), human CD31 (red), and αSMA (white). Scale bar, 100 µm. (E) Quantification of vessel density for hESC-EC vessels (DsRed+, red circles) and all perfused vessels (Lectin+, blue circles) in SA

and μV + SA grafts compared to that in infarct regions. N = 5 biologically independent animals for infarct. Representative images for (A), (B), (C), and (D) from 6 biologically independent animals containing SA grafts, 6 biologically independent animals containing μV + SA grafts, and 12 healthy regions that correspond to all animals containing grafts, with similar results. Hoechst-stained nuclei, blue. Hoechst-stained nuclei, blue. GSL I, *Griffonia simplicifolia* Lectin I. UEA I, *Ulex europaeus* Agglutinin I.

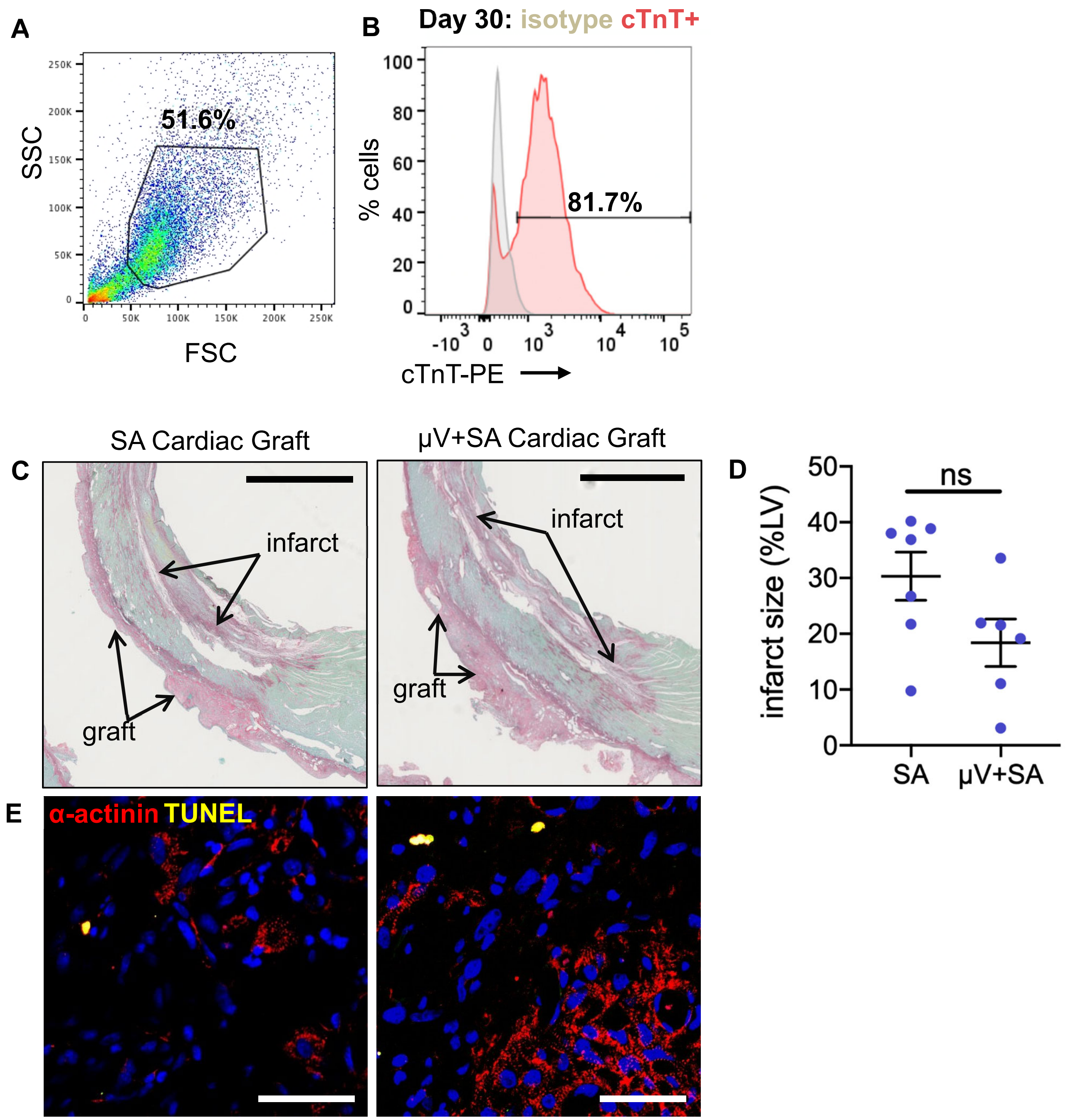

Supplementary Figure 11. Additional analysis of cardiac constructs 5 days post implantation (A-B) Flow cytometry assessment of Day 30 cTnT+ cardiomyocyte population from hESC differentiation. (A) Forward and side scatter used to gate main population. (B) cTnT expression of hESC-CMs with overlay of isotype controls. (C) Picrosirius red/fast green stain to SA (left) and μV + SA (right) grafts. Collagenous regions, such as the infarct and grafts, stain red/purple and healthy tissue regions stain green. Scale bar, 2 mm. (D) Quantification of infarct size as a

percentage of left ventricle area. N = 7,6 biologically independent animals for SA and μV + SA, respectively. $p > 0.05$ (two-tailed t-test). (E) TUNEL assay for apoptotic cells (TUNEL, yellow) on paraffin sections of SA and μV + SA grafts with co-stain for cardiomyocytes (α-actinin, red). Scale bar, 50 μm. Representative images for (C) and (E) from 7 biologically independent animals containing SA grafts and 6 biologically independent animals containing μV + SA grafts, with similar results. Error bars, mean ± SEM. ns = non-significant ($p > 0.05$).
