# Supplementary material for: Patterned human microvascular grafts enable rapid vascularization and increase perfusion in infarcted rat hearts
Source: Nat Commun. 2019 Feb 4;10:584. doi: 10.1038/s41467-019-08388-7 (PMC6362250; doi:10.1038/s41467-019-08388-7)
Supplement: Supplementary file 2 — Reporting Summary [file 41467_2019_8388_MOESM2_ESM.pdf]

nature research

Corresponding author(s): Ying Zheng, Charles E. Murry

Last updated by author(s): 12/19/2018

Reporting Summary

Nature Research wishes to improve the reproducibility of the work that we publish. This form provides structure for consistency and transparency in reporting. For further information on Nature Research policies, see Authors & Referees and the Editorial Policy Checklist.

Statistics

For all statistical analyses, confirm that the following items are present in the figure legend, table legend, main text, or Methods section.

n/a	Confirmed	
☐	☒	The exact sample size (*n*) for each experimental group/condition, given as a discrete number and unit of measurement
☐	☒	A statement on whether measurements were taken from distinct samples or whether the same sample was measured repeatedly
☐	☒	The statistical test(s) used AND whether they are one- or two-sided<br>*Only common tests should be described solely by name; describe more complex techniques in the Methods section.*
☒	☐	A description of all covariates tested
☐	☒	A description of any assumptions or corrections, such as tests of normality and adjustment for multiple comparisons
☐	☒	A full description of the statistical parameters including central tendency (e.g. means) or other basic estimates (e.g. regression coefficient) AND variation (e.g. standard deviation) or associated estimates of uncertainty (e.g. confidence intervals)
☐	☒	For null hypothesis testing, the test statistic (e.g. *F*, *t*, *r*) with confidence intervals, effect sizes, degrees of freedom and *P* value noted<br>*Give P values as exact values whenever suitable.*
☒	☐	For Bayesian analysis, information on the choice of priors and Markov chain Monte Carlo settings
☒	☐	For hierarchical and complex designs, identification of the appropriate level for tests and full reporting of outcomes
☒	☐	Estimates of effect sizes (e.g. Cohen's *d*, Pearson's *r*), indicating how they were calculated

Our web collection on statistics for biologists contains articles on many of the points above.

Software and code

Policy information about availability of computer code

Data collection

• Microscopic histological and immunofluorescent images were taken using NIS Elements Version 5.00.
• RNAseq data was collected on poly-A-enriched samples using Illumina TruSeq.
• Optical microangiography data was collected using propriety software provided by Dr. Ruikang Wang.
• Hamamatsu Nanozoomer whole slide scanner was used to get the whole slide images. The images from the scanner is viewed with NDP software (NDP.view 2.7.25).

Data analysis

• Statistical analysis was performed using Graphpad Prism software version 8.
• Image J (version 2.0.0-rc-65) was used to view and adjust brightness/contrast of the histological and immunofluorescent images and for quantification purposes.
• Histological and immunofluorescent images were taken using NIS Elements Version 5.00.
• RNAseq data was analyzed using R (version 3.4).
• Optical microangiography data was analyzed using propriety software from Dr. Ruikang Wang lab.
• MATLAB (version 9.4) was used for quantification of vessel density and size.
• COMSOL (version 5.3a) was used to generate flow simulation.

For manuscripts utilizing custom algorithms or software that are central to the research but not yet described in published literature, software must be made available to editors/reviewers. We strongly encourage code deposition in a community repository (e.g. GitHub). See the Nature Research guidelines for submitting code & software for further information.

Data

Policy information about availability of data

All manuscripts must include a data availability statement. This statement should provide the following information, where applicable:

- Accession codes, unique identifiers, or web links for publicly available datasets
- A list of figures that have associated raw data
- A description of any restrictions on data availability

Additional information from the study is available from the corresponding authors, Ying Zheng, PhD. (yingzy@uw.edu) or Charles E. Murry, PhD. (murry@uw.edu), upon request. RNA-seq data have been deposited in the Gene Expression Omnibus database under accession code: GSE124314.

Field-specific reporting

Please select the one below that is the best fit for your research. If you are not sure, read the appropriate sections before making your selection.

☒ Life sciences ☐ Behavioural & social sciences ☐ Ecological, evolutionary & environmental sciences

For a reference copy of the document with all sections, see nature.com/documents/nr-reporting-summary-flat.pdf

Life sciences study design

All studies must disclose on these points even when the disclosure is negative.

Sample size	Sample sizes were determined based on pilot studies without rigorous statistical analysis.
Data exclusions	One animal with µV + SA graft was excluded from graft size calculation due to partial graft removal during tissue processing. No other data was excluded.
Replication	6 pilot in vivo batches were performed to ensure reproducible surgical success and high quality and quantitative results in OMAG data acquisition.
Randomization	Experimental groups were assigned randomly in terms of the rat order and construct types for implantation by two independent observers. One observer selected rat order while the other observer selected construct type without knowledge of the other's selection.
Blinding	All data collection and analysis of animal experiments was performed by a blinded observer without knowledge of experimental group. RNAseq analysis was performed by a blinded observer without knowledge of experimental group. All other in vitro experiments were performed without blinding.

Reporting for specific materials, systems and methods

We require information from authors about some types of materials, experimental systems and methods used in many studies. Here, indicate whether each material, system or method listed is relevant to your study. If you are not sure if a list item applies to your research, read the appropriate section before selecting a response.

Materials & experimental systems

n/a	Involved in the study	
☐	☒	Antibodies
☐	☒	Eukaryotic cell lines
☒	☐	Palaeontology
☐	☒	Animals and other organisms
☒	☐	Human research participants
☒	☐	Clinical data

Methods

n/a	Involved in the study	
☒	☐	ChIP-seq
☐	☒	Flow cytometry
☒	☐	MRI-based neuroimaging

Antibodies

Antibodies used

CD34-APC Mouse monoclonal BD 555824 1:4
CD31-PE Mouse monoclonal BD 555446 1:4
cTnT Mouse monoclonal ThermoScientific MS-295 1:100
CD31 Rabbit polyclonal Abcam 28364 1:25, 1:50
VE-cadherin Mouse polyclonal Abcam 7047 1:50
DsRed Rabbit polyclonal Abcam 16667 1:100
GFP Goat polyclonal Abcam 5450 1:400
vWF Sheep polyclonal Abcam 8822 1:100
CD41a Mouse monoclonal BD 555466 2.5 µg/mL

	DsRed Rabbit polyclonal Clontech 632496 1:75<br>alpha-SMA Mouse monoclonal Abcam 7817 1:100<br>GSL I Goat Vector AS-2104 1:200<br>Rhodamine Mouse monoclonal Abcam 9093 1:150<br>CD68 Mouse monoclonal Serotec MCA341/GA 1:100<br>Sarcomeric alpha-actinin Mouse monoclonal Abcam 9465 1:50<br>Beta-MHC Mouse monoclonal hybridoma supernatant ATCC #CRL-2046 full strength
Validation	Each antibody was validated using established positive and negative controls.

Eukaryotic cell lines

Policy information about cell lines

Cell line source(s)	We used RUES2 (Rockefeller University, NIH 0013) human embryonic stem cell line and human bone marrow-derived stromal cells (courtesy of Beverly Torok-Storb laboratory at Fred Hutchinson Cancer Research Center).
Authentication	None of the cell lines were authenticated.
Mycoplasma contamination	Cell lines were not tested for mycoplasma.
Commonly misidentified lines<br>(See ICLAC register)	Neither of our cell lines are on the ICLAC registry.

Animals and other organisms

Policy information about studies involving animals; ARRIVE guidelines recommended for reporting animal research

Laboratory animals	The animals in our study were male athymic nude Sprague-Dawley rats (approximately 250 g – 300 g, 8 weeks of age).
Wild animals	This study did not involve wild animals.
Field-collected samples	This study did not involve samples collected from the field.
Ethics oversight	All animal procedures in this study were approved by the University of Washington Institutional Animal Care and Use Committee (IACUC, protocol #2225-04) and performed in accordance with US NIH Policy on Humane Care and Use of Laboratory Animals.

Note that full information on the approval of the study protocol must also be provided in the manuscript.

Flow Cytometry

Plots

Confirm that:

☒ The axis labels state the marker and fluorochrome used (e.g. CD4-FITC).

☒ The axis scales are clearly visible. Include numbers along axes only for bottom left plot of group (a 'group' is an analysis of identical markers).

☒ All plots are contour plots with outliers or pseudocolor plots.

☒ A numerical value for number of cells or percentage (with statistics) is provided.

Methodology

Sample preparation	Endothelial progenitor cells (day 5) derived from Rues2 embryonic stem cells were dissociated into single cells by treatment of 0.25% trypsin in EDTA, incubated with CD34–APC mouse monoclonal antibody (BD 555824, 1:4) and isotype control APC mouse IgG monoclonal antibody (BD 555751, 1:49) and fixed in 1% paraformaldehyde.<br><br>Endothelial cells derived from Rues2 embryonic stem cells were dissociated into single cells by treatment of Trypsin-EDTA (0.25%) (Thermo Fisher), incubated with CD31–PE mouse monoclonal antibody (BD 555446, 1:4) and isotype control PE mouse IgG monoclonal antibody (BD 555749, 1:49) and fixed in 1% paraformaldehyde.<br><br>For mTm and GFP assessment, endothelial cells were prepared as above but were unstained for GFP assessment and incubated with DsRed rabbit polyclonal antibody (Abcam 16667, 1:100) and isotype control rabbit IgG polyclonal antibody (Cell Signaling 27295, 1:100) then APC-conjugated donkey anti-rabbit antibody (Jackson Cat 74-136-153, 1:200).<br><br>Cardiomyocytes derived from Rues2 embryonic stem cells were dissociated into single cells by treatment of 0.05% typsin in EDTA, fixed with 4% paraformaldehyde and incubated with cTnT mouse monoclonal antibody (ThermoScientific MS-295, 1:100) and isotype control mouse IgG1 antibody (Thermo Fisher 14-4714-85, 1:100) then PE-conjugated goat anti-mouse antibody (Jackson Cat 115-116-072, 1:200).
Instrument	BD FACSCanto II

Software	BD FACSDiva software, version 8.0.1
Cell population abundance	Cell populations for dual fluorescent hESC reporter line were >96% mTm-positive or >98% GFP-positive for all studies.<br>Endothelial progenitor cell (day 5) populations which were 40-80% CD34-positive were cultured further into endothelial cells.<br>Endothelial cell populations which were 92 to 99% CD31-positive were used for transplantation and in vitro studies.<br>Cardiomyocyte populations which were 78-81.7% cTnT-positive were used for transplantation.
Gating strategy	1) The major cell population which was considered as single cells was gated from a forward scatter and side scatter plot.<br>2) mTm populations were gated using the APC channel of the isotype control such that approximately 5.0% of cells were gated as mTm-positive; GFP populations were gated using the FITC channel of the isotype control such that approximately 5.0% of cells were gated as GFP-positive; Endothelial progenitor cell (day 5) populations were gated using the APC channel of the isotype control such that approximately 5.0% of cells were gated as CD34-positive; Endothelial cell populations were gated using the PE channel of the isotype control such that approximately 5.0% of cells were gated as CD31-positive; Cardiomyocytes populations were gated using the PE channel of the isotype control such that approximately 5.0% of cells were gated as cTnT-positive.<br>3) The gate of the isotype control was used to determine population size of mTm-positive cells, GFP-positive cells, CD34-positive cells, CD31-positive cells, or cTnT-positive cells.

☒ Tick this box to confirm that a figure exemplifying the gating strategy is provided in the Supplementary Information.
