# Supplementary material for: Patterned human microvascular grafts enable rapid vascularization and increase perfusion in infarcted rat hearts
Source: Nat Commun. 2019 Feb 4;10:584. doi: 10.1038/s41467-019-08388-7 (PMC6362250; doi:10.1038/s41467-019-08388-7)
Supplement: Supplementary file 13 — Description of Additional Supplementary Files [file 41467_2019_8388_MOESM13_ESM.docx]

**Supplementary Movie Legends**

**Title: Supplementary Movie 1.**
**Description:** Animation of a confocal z-stack image of µV + SA device to demonstrate the extent of integration between endothelial sprouts and de novo formed lumens. DsRed (red, mTm-hESC-ECs), GFP (green, GFP-hESC-ECs), and Hoechst (blue, Nuclei).

**Title: Supplementary Movie 2.**
**Description:** Red fluorescent beads (diameter 1 μm) perfusing through device and clearly filling the endothelial sprouts.

**Title: Supplementary Movie 3.**
**Description:** Whole blood perfusion through hESC-EC seeded device. Individual red blood cells can be seen filling the microvessel before moving into and filling endothelial sprouts.

**Title: Supplementary Movie 4.
Description:** Higher magnification video of red blood cell movement through and endothelial sprout that bridges two sections of the patterned vessel.

**Title: Supplementary Movie 5.**
**Description:** Visualization of platelet accumulation during whole blood perfusions. Platelets are labeled in green (CD41a – FITC).

**Title: Supplementary Movie 6.**
**Description:** Higher magnification video of platelet accumulation in a microvessel sprout during whole blood perfusions. Platelets are labeled in green (CD41a – FITC).

**Title: Supplementary Movie 7.
Description:** Animation of OMAG acquisition (cross section with host myocardium on bottom and graft on top) of SA graft. Volumetric vasculature is colored in red and overlaid with tissue structure.

**Title: Supplementary Movie 8.
Description:** Animation of OMAG acquisition (cross section with host myocardium on bottom and graft on top) of µV + SA graft. Volumetric vasculature is colored in red and overlaid with tissue structure.

**Title: Supplementary Movie 9.
Description:** Cardiac construct containing self-assembled vessels (SA) after 4 days of culture prior to implantation.

**Title: Supplementary Movie 10.**
**Description:** Cardiac construct containing patterned, perfusable microvessels (μV + SA) after 4 days of culture prior to implantation.
